# The Effect of an Additional Structured Methods Presentation on Decision-Makers’ Reading Time and Opinions on the Helpfulness of the Methods in a Quantitative Report: Nonrandomized Trial

**DOI:** 10.2196/29813

**Published:** 2022-04-12

**Authors:** Jan Koetsenruijter, Pamela Wronski, Sucheta Ghosh, Wolfgang Müller, Michel Wensing

**Affiliations:** 1 Department of General Practice and Health Services Research University Hospital Heidelberg Heidelberg Germany; 2 Scientific Databases and Visualization Group (SDBV) Heidelberg Institute for Theoretical Studies gGmbH Heidelberg Germany

**Keywords:** decision-making, health care reports, reading behavior, research methods, eye-tracking, perceived importance, electronic health records, feasibility, quantitative methods

## Abstract

**Background:**

Although decision-makers in health care settings need to read and understand the validity of quantitative reports, they do not always carefully read information on research methods. Presenting the methods in a more structured way could improve the time spent reading the methods and increase the perceived relevance of this important report section.

**Objective:**

To test the effect of a structured summary of the methods used in a quantitative data report on reading behavior with eye-tracking and measure the effect on the perceived importance of this section.

**Methods:**

A nonrandomized pilot trial was performed in a computer laboratory setting with advanced medical students. All participants were asked to read a quantitative data report; an intervention arm was also shown a textbox summarizing key features of the methods used in the report. Three data-collection methods were used to document reading behavior and the views of participants: eye-tracking (during reading), a written questionnaire, and a face-to-face interview.

**Results:**

We included 35 participants, 22 in the control arm and 13 in the intervention arm. The overall time spent reading the methods did not differ between the 2 arms. The intervention arm considered the information in the methods section to be less helpful for decision-making than did the control arm (scores for perceived helpfulness were 4.1 and 2.9, respectively, range 1-10). Participants who read the box more intensively tended to spend more time on the methods as a whole (Pearson correlation 0.81, *P*=.001).

**Conclusions:**

Adding a structured summary of information on research methods attracted attention from most participants, but did not increase the time spent on reading the methods or lead to increased perceptions that the methods section was helpful for decision-making. Participants made use of the summary to quickly judge the methods, but this did not increase the perceived relevance of this section.

## Introduction

Many quantitative reports are produced to help decision-makers in clinical practice, management, and health care policy. For the adequate use of these reports in decision-making, understanding the research methods used to generate their results and conclusions is essential for an assessment of the validity of the presented quantitative data. Previous studies have shown that the methodological limitations of claims made about health interventions are neglected or not well understood by readers [1]. Even policy makers often fail to think critically about the trustworthiness of claims, and many people do not grasp that two things can be associated without one necessarily causing the other [1]. Various studies have shown that clinicians do not completely understand the information on treatment effects from meta-analyses [2] and that midwives and obstetricians are often unable to correctly interpret probabilistic screening information [3]. The QuantEV study examined the reading behavior of potential decision-makers by showing them a quantitative data report and found that reading behavior in the methods sections was variable and that overall, the section was not read thoroughly [4]. Critical assessment of the validity of data and methods is a part of the education of many health professionals and health care decision-makers (as one source puts it: “You cannot judge results without judging methods” [5]). Recently, an alliance of 24 researchers stated that teaching people to think critically about claims and comparisons will help them to make better decisions [1]. As more large-scale data is routinely collected and becomes available for decision-makers, the importance of knowing and understanding the validity and limitations of data reports has increased accordingly.

So far, a considerable body of research has focused on the evaluation and improvement of the critical appraisal skills of research users; in other words, how to train these users to increase their knowledge and improve their attitudes toward the use of research evidence [6]. Multiple studies have addressed the topic of reporting evidence (in this context, this corresponds to reporting results) and have given recommendations on how to present quantitative results visually [7,8]. By contrast, few intervention studies have focused on how research reports should present the validity of the evidence (ie, how they should present the methods). Moreover, many studies on reading behavior have used measurements that depend on self-reporting, or they have used methods like thinking aloud or the click-and-read method, all of which have uncertain validity [9-11]. Thus, we observe a need for readers to pay more attention to the methods used in reports and a lack of studies on how to achieve this. In the light of these observations, we developed an intervention which aimed at enhancing the understanding of the methods used in reports.

To design this intervention, we built on the results of a previous study (QuantEV), which assessed the reading behavior of future health policy decision-makers who were given quantitative data reports [4]. That study showed a high variation in reading time for the methods section, indicating that some decision-makers read the methods well, but others hardly paid attention to them. Reasons for paying less attention varied, but time constraints emerged as an important factor. In particular, the interplay between perceived relevance and time constraints led some participants to spend time on only those sections that they perceived as most relevant to decision-making. Although it is not easy to improve perceptions of relevance and methodological knowledge, time constraints can be addressed by reducing the time necessary to read the methods section.

To test whether reducing the time necessary to read the methods section would increase the attention paid to it, we developed an information textbox that offered a structured summary of a report’s methods section. This textbox was placed at the beginning of the methods section in the upper left position, as this position attract readers’ initial attention [12]. We designed the box while following guidelines developed for designing readable patient education materials. These guidelines state that critical information should be placed prominently, important elements and key points should be highlighted with visual cues (using devices such as boxes), and lists should be bulleted, so that they are easier to follow [13]. The textbox showed the structure of the report with headings that corresponded to the elements that readers usually find first during the skimming phase of reading [14]. We also used appropriate highlighting, which has been shown to enhance comprehension [15] and added tables, which are read more extensively than free text [16]. Drawing attention to a textbox enables readers to quickly judge whether there is anything questionable about the methods. In this way, adding a box can motivate readers to read more of the full methods section. Thus, this study set out to investigate whether adding a box led readers to read the methods more extensively. A secondary aim was to examine how this box influenced readers’ appreciation of the importance of the methods for decision-making.

We hypothesized that by including a box with the highlights of the methods section, more participants would read at least a part of the methods section and the overall attention paid to the methods section would increase.

## Methods

### Study Design

We performed a nonrandomized pilot trial with a historical control arm in a computer laboratory setting. The trial used a computer-based quantitative data report; outcome measures were obtained with an eye tracker, a questionnaire, and a semistructured interview. The aim was to explore whether presenting the methods section in a structured and summarized manner could increase reading time and the perceived importance of the methods section.

### Study Population and Research Setting

The study population was sampled from medical students in their seventh semester of study or later. All were potential future health care professionals who might become involved in local health care policy making. The eye-tracking measurements required that participants were not blind and did not have implanted artificial lenses. For the reading task, we requested that subjects have good knowledge of the German language. Students were invited to join the study with an email that was sent by the study program coordinators or the secretary. Additionally, posters were placed on campus and short presentations were given to students studying for bachelor’s or master’s degrees. For the control group, we used a historical control arm taken from the original project; these participants thus received the standard report. For the intervention group, a new sample was selected following the same criteria as the control group. A total sample size of at least 30 participants was felt to be sufficient for the exploratory nature of this pilot study.

Data were collected at the eye-tracker laboratory of the Scientific Databases and Visualization group at the Heidelberg Institute for Theoretical Studies between April 2019 and March 2020.

### Intervention

Participants were presented a decision scenario in the field of health care policy making with a quantitative data report to support the decision. The scenario was hypothetical, yet realistic: the participants had to advise the local district administrator on the use of additional funds for long-term care. The participants had to choose from 3 predefined options given by the research team. They were instructed to spend no more than 20 minutes on both reading the report and making the decision. We assumed a reading speed of 5 minutes per 1000 words, so given that the report contained around 4400 words, this time constraint was tight [17]. This was a deliberate choice, as in real decision-making scenarios, the amount of available information exceeds the time to read all of it. We also wanted to force the participants to restrict their reading time to the report sections that they found most valuable, thereby revealing how they prioritized the sections. The presented report was written in German and was 13 pages long, containing 3915 words. It was structured as a short project report, comprising a title page, a table of contents, an introduction (together approximately 1.5 pages), a methods section (approximately 3.5 pages), a results section (approximately 4.5 pages), and a discussion and conclusion section (together approximately 1 page). The quantitative data presented in the report were real descriptive figures on the current and projected demand and supply of long-term-care services in the region of interest; the data were based on secondary data analyses of real data [18].

**Figure 1:**
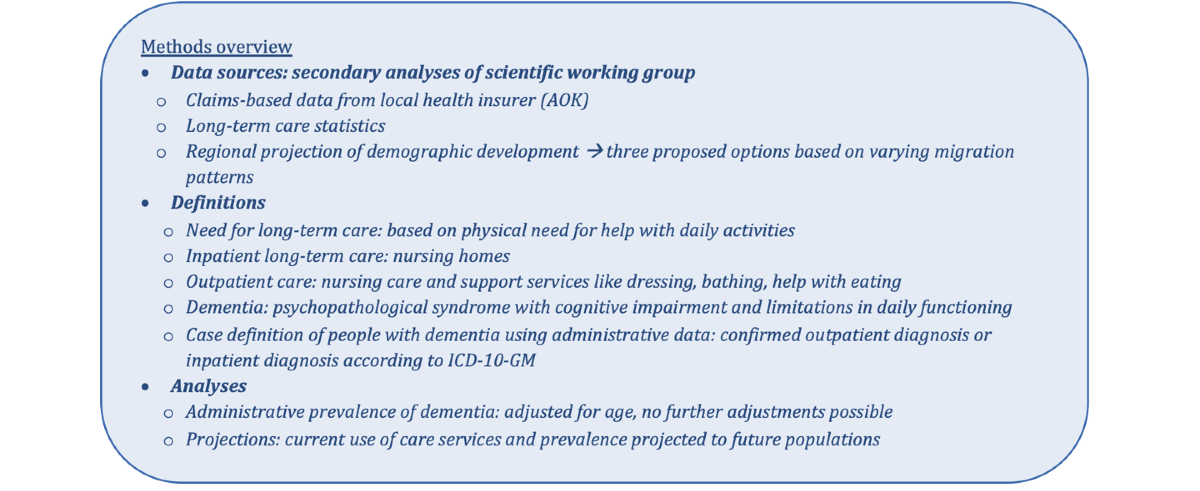
Example of textbox showing highlights of the study methods.

The historical control arm received the original report with a traditional methods section, whereas the intervention arm received a version of the report with an additional textbox containing highlights of the methods. This box was developed to offer a structured presentation of the key aspects of the study design and methods. We designed the box to follow the STROBE (Strengthening the Reporting of Observational Studies in Epidemiology) checklist for conference abstracts, as provided by the Equator Network [19]. Thematically related bullet points were presented following the original structure of the full methods section (ie, data sources, definitions, and analyses) (Figure 1). Thus, this box provided the most relevant information at a glance and could help readers quickly grasp key information about the methods.

### Measurements

We collected 3 different types of measurements from each participant: eye-tracking data during the performance of the task, answers to a questionnaire, and findings from a face-to-face, semistructured interview conducted after the task. For the eye tracking, we used the Tobii-X1 Light (Tobii AB) [20], a desktop-mounted, binocular eye tracker, and the Tobii eye-tracker software (version 3.4.8).

Based on the eye-tracking data, we extracted 3 measurements and calculated their mean values for the methods section (which included the box in the intervention group). First, we recorded the time spent (in minutes) to read the report and complete the task. Second, we computed the average fixation duration (in milliseconds) with the Tobii I-VT fixation filter and Tobii eye-tracker software [21]. Fixation duration was used as an indicator of attention to the report and information processing by the reader [22]. Fixation duration for individual readers varies during reading, and this variability can be used to provide a real-time reflection of ongoing cognitive and language processing [22]. Fixation duration serves as a primary source of evidence for testing theories of reading, and the central focus of computational models of skilled reading is to account for the influence of text processing on fixation duration [23-25]. Third, pupillary response was measured as an indicator of cognitive load [26-28]. The pupillary response was calculated as the change in pupil diameter from the average value as the eye passed over the screen [29]. While fixation duration accounts for cognitive and language processing, the pupillary response is associated with several cognitive functions, such as mental effort, interest, and decision-making [30-32]. All these cognitive functions, which cause variation in the size of the pupil, are related to attention [33,34]. Our main hypothesis was that the time spent on reading the methods section would be higher in the intervention group. Furthermore, we expected that attention would be higher in the intervention group.

The questionnaire was used to collect individual characteristics, such as age, sex, and the participants’ risk literacy. Risk literacy was measured using the validated Berlin numeracy test, which is a 4-item paper and pencil test taken in the German language [35]. We expected that participants with higher risk literacy would have more affinity with the quantitative methods. Risk literacy was therefore measured to control for potential differences between study arms. Finally, the participants were asked to assess each section of the report (ie, the introduction, methods, results, discussion, and conclusion) for understandability and helpfulness during the decision-making task, both on a 10-point Likert scale.

A semistructured question guide was developed to explore the experiences of the participants in completing the task. Interview questions were developed by the authors in cooperation with 2 colleagues with a sociology and health science background. The participants were encouraged to speak frankly about their experiences generally and about the way they had read the report specifically. The interviews were audio recorded and transcribed. Additional details on these methods have been previously published [4].

### Analyses

Participants were the unit of analysis. Questionnaire and eye-tracking data (after data preparation with the Tobii eye-tracker software) were analyzed using SPSS Statistics (version 25; IBM). Descriptive analyses and the *t* test were used to find differences between the study arms. Additionally, the Pearson correlation was calculated to explore the relationships between fixation, pupillometric data, and participant characteristics. Considering the exploratory nature of the study and the small sample size, we regarded a *P* value <.1 as significant. For analyzing the interviews, qualitative content analysis was conducted to explore reasons mentioned by participants as to why they gave more or less attention to a report section during decision-making. The qualitative content analysis was conducted by 2 of the authors with the support of a third colleague.

### Ethics Approval

This study was approved by the research ethics committee of Heidelberg University Hospital (S-857/2018) and was part of the QuantEV project, which has been described previously [4]. Before data collection, participants were informed orally and in writing about the study context, the data collection procedure, and data security. All participants provided consent for participation. Participation was voluntary, and study withdrawal was possible at any time before the collected data were anonymized.

## Results

In total, 35 participants were included, 22 in the control arm and 13 in the intervention arm (Table 1). In both the control and intervention arms, women were more represented (18/22, 82%; and 8/13, 64%, respectively). The average age of the participants was 23.7 years; this was similar between the arms. The average risk literacy score was 68.0 (ie, 68% of the answers were correct); this was similar between the control and intervention arms (with an average risk literacy score of 69.3 and 65.4, respectively). The decisions made after reading the report were also similar in both groups, with option C (increasing ambulant nursing capacity) being the dominant choice, chosen by 27 of 35 (77%) of the participants.

**Table 1:** Characteristics of study participants.

Characteristics		Control group (n=22)	Intervention group (n=13)	Overall (N=35)
Female, n (%)		18 (82)	8 (64)	27 (76)
Age (years), mean (SD)		23.9 (1.5)	23.5 (2.3)	23.7 (1.8)
Risk literacy score, mean (SD)		69.3 (33.6)	65.4 (31.5)	68.0 (32.4)
**Decision on how to spend funds**				
	Option A: more support for informal caregivers, n (%)	2 (9)	2 (15)	4 (11)
	Option B: more nursing home capacity, n (%)	3 (14)	1 (8)	4 (11)
	Option C: more ambulant nursing capacity, n (%)	17 (77)	10 (77)	27 (77)

Table 2 shows the eye-tracking and questionnaire results for participants from both study arms and eye-tracking data for the intervention group only. Differences between the study arms were tested for statistical significance (rightmost column). The overall time spent on the methods and the pupillary response did not differ between the 2 study arms. The average fixation duration was higher in the control arm, but not significantly (0.445 seconds vs 0.337 seconds, *P*=.56). The questionnaire results did not show a difference in perceived understandability, but the intervention group found the methods less helpful for decision-making (score 2.9 vs 4.1, *P*=.09). These findings do not provide support for the hypothesis that the box would increase attention paid to the methods section. The box-only results for the intervention group showed that the participants spent about half a minute reading the box, while the other eye-tracking values were similar to those for the overall methods.

**Figure 2:**
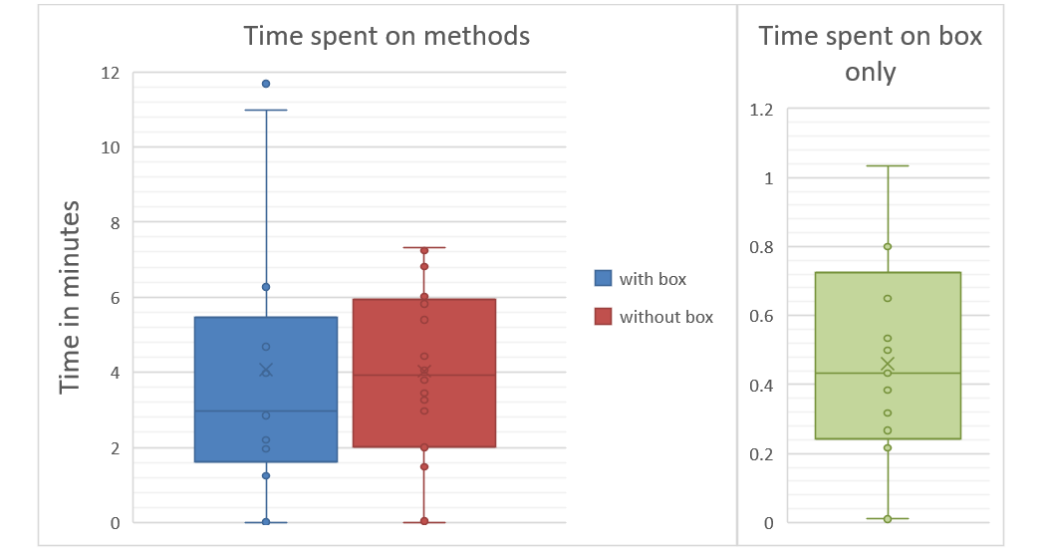
Time spent reading the methods section in reports with and without an added box (in the intervention and control groups) and time spent reading the box itself (in the intervention group) in minutes.

Figure 2 shows box plots for time spent on reading the methods section in both study arms. Whereas the mean time was very similar, the median time, as well as the 25th and 75th percentiles, were lower in the intervention arm. The variation was also higher in the intervention group, mostly due to 2 participants who spent over 10 minutes reading the methods. Our findings for variation in time spent looking at the box showed that 2 participants hardly looked at it at all, while the other participants spent between 0.2 and 1 minute looking at it. Most of the participants took at least a brief look at the box, which could provide support for our hypothesis that the box would lead to more participants reading at least a minimal part of the methods section.

**Figure 3:**
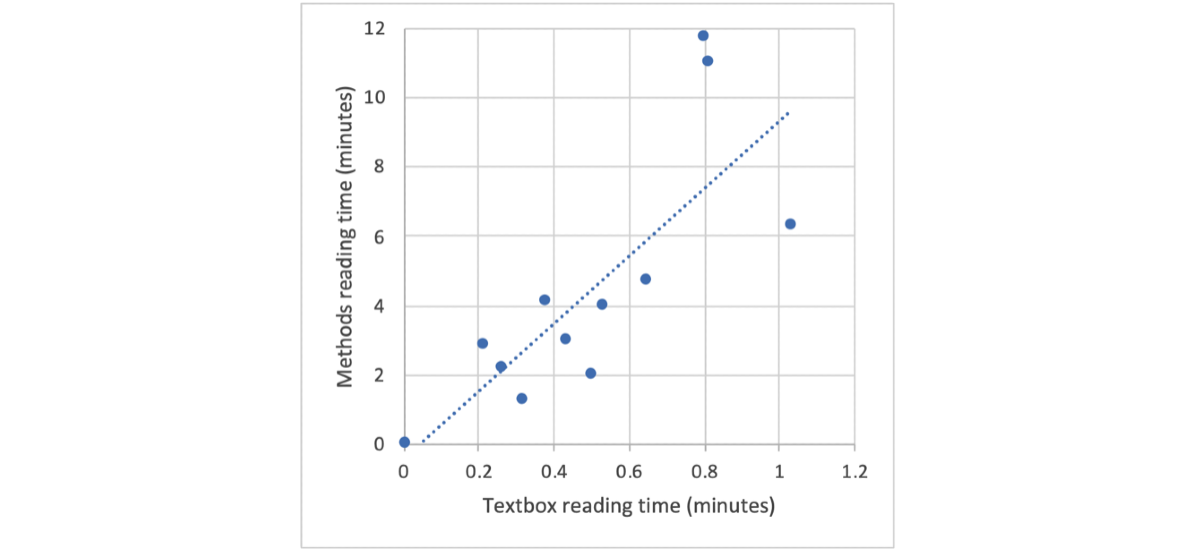
Time spent on reading the box vs the full methods.

Figure 3 shows the relationship between the time spent reading the box and the time spent subsequently reading the whole methods section in the intervention group. Each data point represents a single participant. There was a clear positive relationship between the 2 parameters: participants who spent more time reading the box tended to spend more time reading the full methods (Pearson correlation 0.81, *P*=.001). This relationship held for both the single participant who did not read either the box or the full methods and for the 3 participants who spent the most time on the box, who were also the ones who spent the most time on the methods.

**Figure 4:**
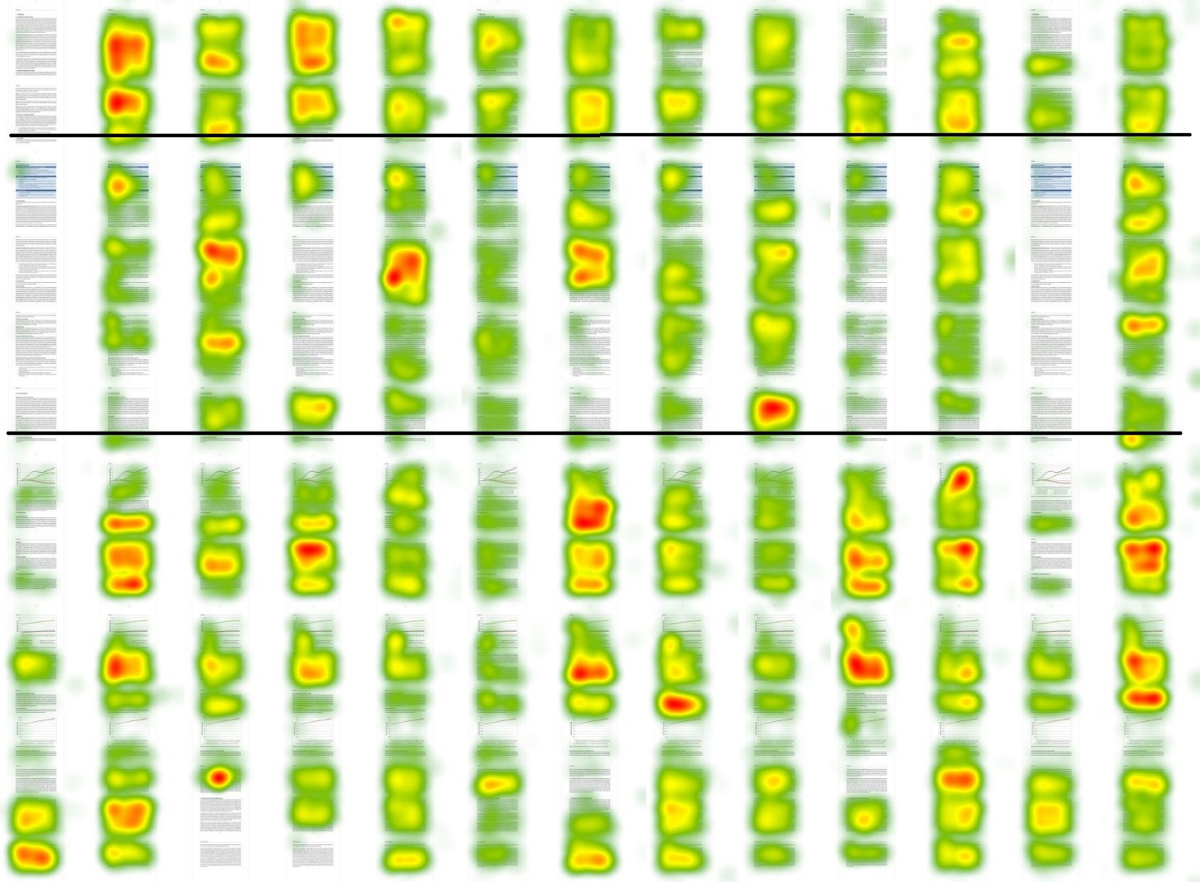
Heat map of intervention group reading time. The methods section is shown between the horizontal lines.

We used heat maps to perform a qualitative analysis of reading patterns. This confirmed the relationship between time spent reading the box and the full methods (Figure 4). Participants who read the box spent more time reading the methods section. In some cases, a participant only briefly skimmed the box and ignored the rest of the methods. This provides support for the idea that introducing the box prompted participants to read at least a minimal amount of the methods section. There was no case in which a participant read the box thoroughly and then skipped reading the full methods. This supports the idea that the box could potentially increase overall attention paid to the methods section. Some participants read the whole methods section, including the box, while others paid more attention to specific subsections.

**Table 2:** Eye-tracking and questionnaire measures by study arm.

Measure		Control	Intervention			*P* value Δ: control – intervention
		Overall	Overall	Box only		
**Eye tracking**						
	Time spent in minutes, mean (SD)	4.03 (2.39)	4.07 (3.68)	0.46 (0.31)		.96
	Average fixation duration in seconds, mean (SD)	0.445 (0.643)	0.337 (0.213)	0.316 (0.145)		.56
	Pupillary response in mm, mean (SD)	0.033 (0.011)	0.034 (0.017)	0.032 (0.014)		.91
**Questionnaire**						
	Understandability, range 1-10, mean (SD)	6.6 (2.1)	6.6 (2.0)		N/A	.96
	Helpfulness for decision-making, range 1-10, mean (SD)	4.1 (2.2)	2.9 (1.3)		N/A	.09

^a^N/A: not applicable.

In the qualitative interviews, participants reported why they preferred specific report sections. Some participants reported that they paid less attention to the methods because they did not perceive them as relevant for decision-making, and that they trusted the authors to have used valid methods. Other participants reported that they used the box to gain a broad understanding of the methods, and then only briefly scanned the full section, because they did not perceive it as very important and felt it was not necessary to read it thoroughly.

## Discussion

Adding a textbox with a structured summary of the methods did not increase the total time spent reading the full methods section, but it was successful in attracting attention, as most participants at least skimmed the box. However, including the box resulted in a lower appreciation of the helpfulness of the information on research methods. Participants who spent more time on the box also spent more time on the methods in general. The finding that the box seemed to attract attention provides support for our hypothesis that it led more participants to read at least a minimal portion of the methods section. Our findings from the heat map also support this hypothesis. However, as overall reading time did not increase, and we even found that appreciation of the helpfulness of the methods section decreased, we did not obtain support for our hypothesis that the box would increase the overall attention paid to the methods section.

Hypothetically, including the box could have either enhanced or reduced time spent reading the methods section. If it had been seen as complementary information, as we hypothesized it would be, it could have motivated participants to read the full text. However, the finding that there was no increase in overall reading time for the methods does not support the idea that the box was complementary. The linear relationship between time spent reading the box and the methods could have been caused by general interest, or lack thereof, in the methods, rather than indicating that participants read the box purposefully, to quickly gain an overview of the methods without having to read the full section. A potential explanation was provided by the interviews: some participants indicated that they paid less attention to the methods because, considering the time constraints, they did not perceive this section as relevant. The specific sample of medical students examined in this study could also have been a factor, as they might not have been very comfortable with quantitative and statistical methods [36]. Thus, rather than serving a complementary function, the box could have been used as a substitution for the full text, resulting in less attention paid to the methods as a whole. This explanation is supported by the findings that participants did not spend less time on the methods overall and that there was a positive, linear relationship between time spent reading the box and the methods.

The finding that fixation duration was shorter in the intervention group (although this was nonsignificant) could indicate that there was less engagement with the presented text [22]. This explanation would correspond with the finding that by adding the box, the methods were perceived as less useful to complete the presented task. However, fixation duration could also indicate language processing [37]. If the box helped the reader to become familiar with the topic, the full methods section might have been perceived as less complex, reducing the need for language processing and enabling an increase in reading speed. Our use of eye tracking in addition to a questionnaire allowed us to collect rich data on reading behavior that was not influenced by the limitations of self-reported behavior. Limitations in our study were caused by the specific participants we recruited and our measurements of reading time. Our sample consisted of future health care professionals with only limited experience in decision-making, meaning that the findings may not be fully generalizable to more experienced policy makers, who might have perceived and used the report differently. However, our participants were already advanced students and all had received training in interpreting studies, reflected by a slightly higher numeracy level than general practitioners and other medical students [38]. Our method of measuring time spent on the methods did not automatically mean that a subject also read the text. Nevertheless, our findings on average fixation duration suggest that our subjects did read the text, as fixation is, on average, about 0.25 seconds while reading [22]. Additionally, the study design was primarily tailored to the design of a past project rather than to the present intervention study.

In this study, we aimed to explore whether presenting the methods of a report as a structured summary could increase time spent reading the methods section. Our findings indicate that including a box might help to attract attention, but that it might not increase overall interest in the methods section. The intervention might have motivated more decision-makers to read at least some of the methods and helped them judge if the methods needed a full inspection. However, the limited attention paid to the methods by some participants, who considered the methods not relevant for decision-making, is a problem that might not be solvable by changing the input (ie, the format of the report). Rather, it might require an intervention at the individual level to increase awareness of the relevance of the methods section to decision-making.

## Data Availability

The data sets used and analyzed during the current study are available from the corresponding author on reasonable request.
